# Humboldt’s legacy: explaining the influence of environmental factors on the taxonomic and phylogenetic diversity of angiosperms along a Neotropical elevational gradient

**DOI:** 10.1093/aobpla/plac056

**Published:** 2022-11-07

**Authors:** Jorge Antonio Gómez-Díaz, César Isidro Carvajal-Hernández, Alma Patricia Bautista-Bello, María Leticia Monge-González, Valeria Guzmán-Jacob, Holger Kreft, Thorsten Krömer, Fabricio Villalobos

**Affiliations:** Centro de Investigaciones Tropicales, Universidad Veracruzana, 91000 Xalapa, México; Biodiversität, Makroökologie und Biogeographie, Faculty of Forest Sciences and Forest Ecology, University of Göttingen, 37077 Göttingen, Germany; Instituto de Investigaciones Biológicas, Universidad Veracruzana, 91190 Xalapa, Mexico; Department of Biology and Environmental Sciences (IBU), Carl von Ossietzky Universität Oldenburg, 26129 Oldenburg, Germany; Biodiversität, Makroökologie und Biogeographie, Faculty of Forest Sciences and Forest Ecology, University of Göttingen, 37077 Göttingen, Germany; Biodiversität, Makroökologie und Biogeographie, Faculty of Forest Sciences and Forest Ecology, University of Göttingen, 37077 Göttingen, Germany; Biodiversität, Makroökologie und Biogeographie, Faculty of Forest Sciences and Forest Ecology, University of Göttingen, 37077 Göttingen, Germany; Centre of Biodiversity and Sustainable Land Use, University of Goettingen, 37077 Göttingen, Germany; Centro de Investigaciones Tropicales, Universidad Veracruzana, 91000 Xalapa, México; Laboratorio de Macroecología Evolutiva, Red de Biología Evolutiva, Instituto de Ecología A.C., 91073 Xalapa, Mexico; Departamento de Ecologia, Instituto de Ciências Biológicas, Universidade Federal de Goiás, 74690-900 Goiânia, Brazil

**Keywords:** Angiosperms, forest ecology, forest-use intensity, Mexico, Neotropics, phylogenetic structure, structural equation modeling, temperature

## Abstract

The scientific work of Alexander von Humboldt was influenced by his interaction with the diversity and natural wealth of the Neotropics. He proposed that climate determines plant diversity along elevational gradients based on his observations. Here, we evaluated the most prominent climate-based hypotheses in explaining plant diversity along an elevational gradient that Humboldt himself visited during his journey across Mexico. Specifically, we examined how climatic variables and forest-use intensity affected species richness and phylogenetic structure of major angiosperm life forms (trees, shrubs, epiphytes, herbs and lianas) along the Cofre de Perote mountain, Veracruz, Mexico. We analysed species richness and phylogenetic structure of angiosperms at eight sites between 30 to 3500 m a.s.l. We estimated the phylogenetic structure using a mega-phylogeny of angiosperms and the abundance-weighted net relatedness index. We considered multiple environmental factors’ direct and indirect effects by applying a piecewise structural equation modelling approach. Each life form responds differently to the environmental variables included in our model; however, it is observed that temperature is the main predictor of the taxonomic and phylogenetic diversity of the angiosperms studied, both when the different life forms are grouped and separated. Potential evapotranspiration and precipitation are variables that also influence some life forms’ diversity, especially taxonomic diversity. The forest-use intensity negatively affected only the taxonomic diversity of trees. These results highlight the influence of studying the different life forms of angiosperms in diversity gradient models and show the great influence that temperature has in conjunction with other environmental variables to promote the taxonomic and phylogenetic diversity of plant communities. Given the current global environmental crisis, an integrative biogeographically oriented vision based on Humboldt’s method is necessary. Honouring the work of Humboldt and continuing his legacy demands more research to understand the causes behind elevational diversity gradients.

## Teaser

The scientific work of Alexander von Humboldt was influenced by his interaction with the diversity and natural wealth of the Neotropics. Here, we evaluated several hypotheses in explaining plant diversity along an elevational gradient that Humboldt himself visited during his journey across Mexico. We analysed the species diversity of flowering plants at eight sites between 30 to 3500 m a.s.l. We found that temperature was the main predictor of the diversity of the studied plants. Given the current global environmental crisis, an integrative vision based on Humboldt’s method is necessary.

## Introduction

The scientific work of Alexander von Humboldt was influenced by his interaction with the diversity and natural wealth that he observed during his journey through the Neotropics (Humboldt 1807). He travelled through Mexico for about 1 year, where he visited the state of Veracruz and ascended the summit of Cofre de Perote (Humboldt 1811). Later, with the information gathered on his travels, he made the first comparative drawings where the change of plant species across elevation bands is described. Humboldt and colleagues’ work followed an individualistic approach where each species has its place along the elevational gradient (Schrodt *et al*. 2019). This approach is akin to a Gleasonian view—the plants found in a site are a set of species that individually interact with their environment—and is even a precursor of this view (Nicolson and McIntosh 2002; Schrodt *et al*. 2019).

Humboldt’s legacy continues today, especially regarding the knowledge about biodiversity patterns and their causes that can be obtained from studying elevational gradients (e.g. Morueta-Holme *et al*. 2015). Humboldt, a German geologist, and naturalist, and Aimé Bonpland, a French botanist, proposed that climate regulates plant community composition and diversity, as exemplified in their observation of changes in the richness and composition of plants along elevational gradients in the mountains (Humboldt and Bonpland 1805). This observation has influenced the study of ecological gradients, as evidenced by the existence of several hypotheses explaining species richness along elevational gradients as a function of climate (Rahbek *et al*. 2019).

Elevational gradients are considered natural laboratories because studying environmental variables in an elevational gradient confers a methodological advantage by containing in a short distance ample climatic variation, which would otherwise be difficult to capture. An elevational gradient makes it possible to evaluate an environmental gradient of that magnitude, in a smaller and more controlled area, something that Humboldt himself elucidated in his works (Rahbek *et al*. 2019). Different climate-centred hypotheses have been proposed to explain elevational diversity gradients (Graham *et al*. 2014), mostly mirroring those raised for latitudinal diversity gradients such as productivity, precipitation and temperature hypotheses (Hawkins *et al*. 2003).

Humboldt’s influence on biogeography remains strong, especially in explaining the distribution of species due to its relationship with climate in elevational gradients. In general, he made the first empirical observations and descriptions of the relationships between environmental conditions and vegetation on gradients (Humboldt and Bonpland 1805). He is even known as the first to describe how climatic conditions in conjunction with human influence determine plant occurrences and vegetation (Wilson 1995). All this culminated in the development of the hypothesis of the water–energy dynamics (O’Brien *et al*. 2000), which is one of the hypotheses that explain the diversity along elevational gradients, considered one of the most outstanding patterns in biogeography, which is still valid but also subject to discussion (Kinlock *et al*. 2018; Schrodt *et al*. 2019).

Distinct taxonomic groups or life forms can respond similarly or differently to environmental variation along elevational gradients (Peters *et al*. 2016). For example, different plant life forms deal with such environmental variation in distinct ways, and thus considering only one group or lumping species from diverse groups could lead to an incomplete or biased view of general patterns (Vázquez and Givnish 1998). Using a multi-taxon/group approach can help avoid this bias, foster our understanding of elevational gradients and reveal the generality (or lack thereof) of different hypotheses for such gradients (Peters *et al*. 2016).

Moreover, considering biodiversity facets beyond species richness, such as phylogenetic diversity, can further help to reveal evolutionary and ecological processes determining elevational gradients (Graham *et al*. 2014). The current availability of phylogenetic information for large plant clades allows including such information to study species richness patterns (Qian and Jin 2016). Studying phylogenetic diversity is important for conservation biology during the climate change and biodiversity crisis that we are living in (Rodrigues and Gaston 2002). Also, the phylogenetic structure can be used to reveal patterns related to phylogenetic niche conservatism (PNC), which is a recognized pattern where closely related species share a similarity in their ecological niches (Wiens and Graham 2005).

Almost 200 years ago, Humboldt described how diversity changes along elevational gradients, even mentioning how human actions affect biogeochemical cycles, which have a long-term influence on biodiversity (Schrodt *et al*. 2019). However, a human-influenced variable is rarely included to explain diversity patterns in an integrated manner with climatic variables (Aufdenkampe *et al*. 2011). Despite its importance, very few studies have analysed the impact of anthropogenic disturbances on biodiversity along elevational gradients (Kessler 2001; Zhang *et al*. 2013; Carvajal-Hernández *et al*. 2017). Land-use change and land-use intensification are modifying the Neotropical forests (Poorter *et al*. 2016), having a negative influence on species richness (Peters *et al*. 2019), especially along elevational gradients (Nogués-Bravo *et al*. 2008).

This study was conducted along the Cofre de Perote elevational gradient, which is situated in the transition zone of two biogeographic regions: the Nearctic and Neotropical. We aim to identify the dominant environmental factors structuring plant diversity and how the relative importance of these factors changes with elevation and forest-use intensity. In addition, we compared these effects among five different plant life forms (trees, shrubs, epiphytes, herbs and lianas). The objective of this study was to evaluate the main existing hypotheses to explain the patterns of diversity in elevational gradients using a complete gradient that spans from the coast to the mountains.

To our knowledge, this study is the first in analysing the effects of climate and anthropogenic disturbance on different plant life forms’ taxonomic and phylogenetic diversity along an elevational gradient. We hypothesize that plant species richness (a) shows an increase with temperature, (b) peaks at high precipitation values and (c) decreases with increasing forest-use intensity. We further hypothesize phylogenetic structure to reveal that (d) close relatives are more common at low temperatures, indicating phylogenetic clustering because of environmental filtering (Qian *et al*. 2017) by selecting related species that can tolerate these conditions, whereas (e) more distantly related species are more common at elevated temperatures, showing phylogenetic overdispersion because of high interspecific competition. Finally, we hypothesize that (f) forest-use intensity will also negatively affect phylogenetic structure with an increase of clustering at higher forest-use intensity.

## Materials and Methods

### Study area

**Figure 1.**
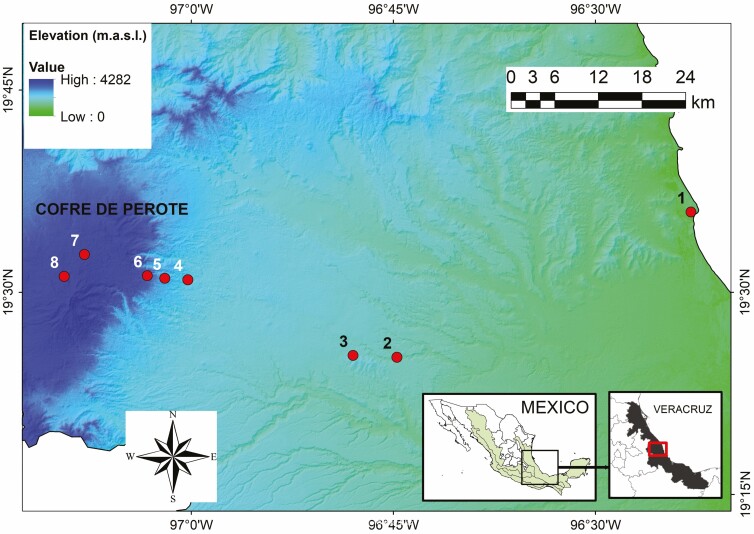
Study area along the elevational gradient at the eastern slopes of the Cofre de Perote mountain, Veracruz, Mexico. Dots show the location of the eight sites.

We established eight sites along an elevational gradient between 30 and 3500 m along the eastern slopes of Cofre de Perote mountain, an extinct volcano of 4282 m in elevation in Veracruz, Mexico (Gómez-Díaz *et al*. 2017). We placed our study locations at constant intervals in the following elevations: 30, 650, 1000, 1500, 2100, 2500, 3100 and 3500 m. Along a straight line of approximately 81 km, the gradient covers six vegetation types (tropical semi-deciduous, tropical oak, humid montane, pine-oak, pine and fir forest; Fig. 1). Our study area presents a wide variety of climates (i.e. warm-dry environments in the lower part, temperate-humid in the mid-montane areas and cold-dry in the upper part; Soto-Esparza and Giddings 2011). Both the temperature and precipitation vary according to elevation. Temperature shows a linear decrease with increasing elevation, whereas average precipitation is higher in the middle parts and decreases at the extremes of the gradient (Carvajal-Hernández *et al*. 2020).

### Sampling

We considered two gradients: the actual elevational gradient from 30 to 3500 m a.s.l. and a disturbance gradient considering three habitats subjected to three different ordinal levels of forest-use intensity: old-growth (OG), degraded (DE) and secondary forest (SE) stands (Gómez-Díaz *et al*. 2017). Old-growth forests were defined as mature forests with a dominance of mature trees in which shrubs cover less than 30 % of the area, with a long time of forest ageing without human disturbance or forest utilization (50 years; Alrutz *et al*. 2022). Degraded forests (DE) are those that still maintain the structure of a conserved forest with remnant trees but are subject to moderate anthropic influence. Secondary forests (locally known as acahuales) were forests not used since clear-cut, controlling a regenerating age between 15 to 25 years after abandonment (based on interviews with local landowners; Alrutz *et al*. 2022).

We established five 20 m × 20 m plots to record the presence (incidence data) of all angiosperm plant species without considering seedlings. The distance among the five plots was in all cases larger than 300 m to avoid any spatial autocorrelation problem. The five plots were nested at each level of forest-use intensity and the three levels of forest-use intensity were nested in each elevational site resulting in a total number of 120 plots (five plots × three habitats × eight elevational sites) and a sampled area of 48 000 m^2^. For details about the vegetation types, study design and forest-use intensity definitions, see Gómez-Díaz *et al*. (2017) and Carvajal-Hernández *et al*. (2020). We obtained climatic variables, mean annual temperature (MAT), and mean annual precipitation (MAP), for each elevational site from an updated, high-resolution (90 m) monthly climate surface for Mexico (Cuervo-Robayo *et al*. 2014) and potential evapotranspiration (PET), from the MODIS/Terra Net Evapotranspiration 8-Day L4 Global 500 m Version 6 (**see****Supporting Information—Fig. S1**; Running *et al*. 2017).

### Species richness and phylogenetic structure

We estimated species diversity as species richness (Hill number *q* = 0) in terms of effective species numbers (Jost 2006). Species richness gives equal weight to common and rare species. To estimate the phylogenetic structure of species in each plot, we obtained information on their phylogenetic relationships from a recently published species-level mega-phylogeny of seed plants (Smith and Brown 2018). This mega-phylogeny is a synthetic tree combining DNA sequences and published phylogenies that includes divergence times. Smith and Brown (2018) provided four large phylogenies, from which we used the ALLMB phylogeny constructed with GenBank, and Open Tree of Life taxa based on a backbone provided by Magallón *et al*. (2015). According to Smith and Brown (2018), this ALLMB phylogeny has fewer conflicts, higher monophyly and broader species sampling, favouring our studied species’ coverage. We pruned the ALLMB phylogeny to include only the species recorded in our gradient.

Despite its broad coverage, several species recorded in our gradient were absent from the mega-phylogeny. To include these missing species, we grafted them into the phylogeny using the function ‘add.species.to.genus’ from the ‘phytools’ package in R (Revell 2012). In short, the imputation proceeds by adding a missing species at the node representing the most recent common ancestor of species from the same genus (Oswald *et al*. 2016).

We obtained the abundance-weighted net relatedness index (NRI), which is defined as the difference in mean pairwise distance (MPD) between observed and randomly generated null communities, standardized by the standard deviation of phylogenetic distances in the null communities (Webb 2000). We created the null communities using the independent swap algorithm (Gotelli 2000), which randomizes the community data matrix maintaining species occurrence frequency and sample species richness, thus randomizing phylogenetic relationships among species, with 1000 iterations. A positive NRI indicates phylogenetic clustering, whereas a negative NRI indicates phylogenetic overdispersion (Qian *et al*. 2019). We used the values of NRI (phylogenetic structure) of each plot for the following step.

### Structural equation models

**Figure 2.**
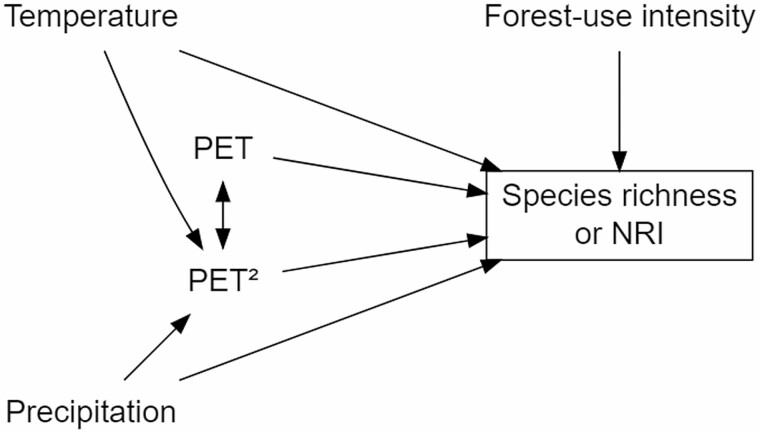
*A priori* structural equation model was used to evaluate environmental effects across plant life forms. Forest-use intensity (a factor with three levels: low, mid and high), temperature, precipitation, potential evapotranspiration (PET) and a quadratic term of potential evapotranspiration (PET^2^).

Species richness and NRI of angiosperm communities may be strongly affected by climatic factors, either directly or indirectly through another variable (Qian *et al*. 2016). To explicitly consider such direct and indirect effects of multiple environmental factors, we applied a structural equation modelling approach based on a hypothetical pathway. In this hypothetical pathway, we considered climate (temperature: MAT and precipitation: MAP), forest-use intensity (a three-step ordinal scale: secondary forest > degraded forest > old-growth forest), and PET, and its quadratic form (PET^2^). The quadratic value of PET^2^ was included as suggested by Vetaas *et al*. (2019), which in turn is based on the water–energy dynamics hypothesis proposed by O’Brien (2006), being how this variable directly influences the diversity of plants in elevational gradients. These variables have all been suggested to directly affect the diversity of angiosperms (Vetaas *et al*. 2019). In sum, we modelled that water (precipitation) and energy (temperature) affect PET and related to the forest-use intensity; they have a direct or indirect effect on the dependent variables (species richness or NRI). With these premises, an *a priori* model was built (Fig. 2).

We fitted piecewise structural equation models (SEM) using the *piecewiseSEM* R package (Lefcheck 2016) separately for each diversity measure (species richness and NRI). Since any parameterization will be taxa-specific, we fitted a piecewise SEM for each plant life form. We used piecewise SEM because we fitted generalized linear mixed models with a Poisson distribution in the case of species richness and Gaussian distribution in the case of NRI. We included the site as a random variable in our SEM models since changes in plant diversity can result from two processes: the effects of the environmental variables included in this study, and the intrinsic difference in species pool among the plots located at different elevations due to the design of the study. Therefore, by including the random effect of the site, we ensure that the explained variance is due solely to the effects of environmental variables and not to a combination between these variables and the species pools. To make the model coefficients comparable, we scaled the variables ($\bar{x}$= 0 and *σ* = 1) before the analysis. All statistical analyses were performed using the statistical software R v.3.6.3 (R Core Team 2020).

## Results

We recorded a total of 689 species in our study area distributed in five different life forms of angiosperms: (i) trees (153 species), (ii) shrubs (123), (iii) terrestrial herbs (229), (iv) epiphytes (164) and (v) lianas (20 species). The taxonomic and phylogenetic richness of the grouped and separated life forms of angiosperms followed different patterns along the elevational gradient **[see****Supporting Information—Figs S2****and****S3****]**.

### Direct and indirect environmental effects

**Figure 3.**
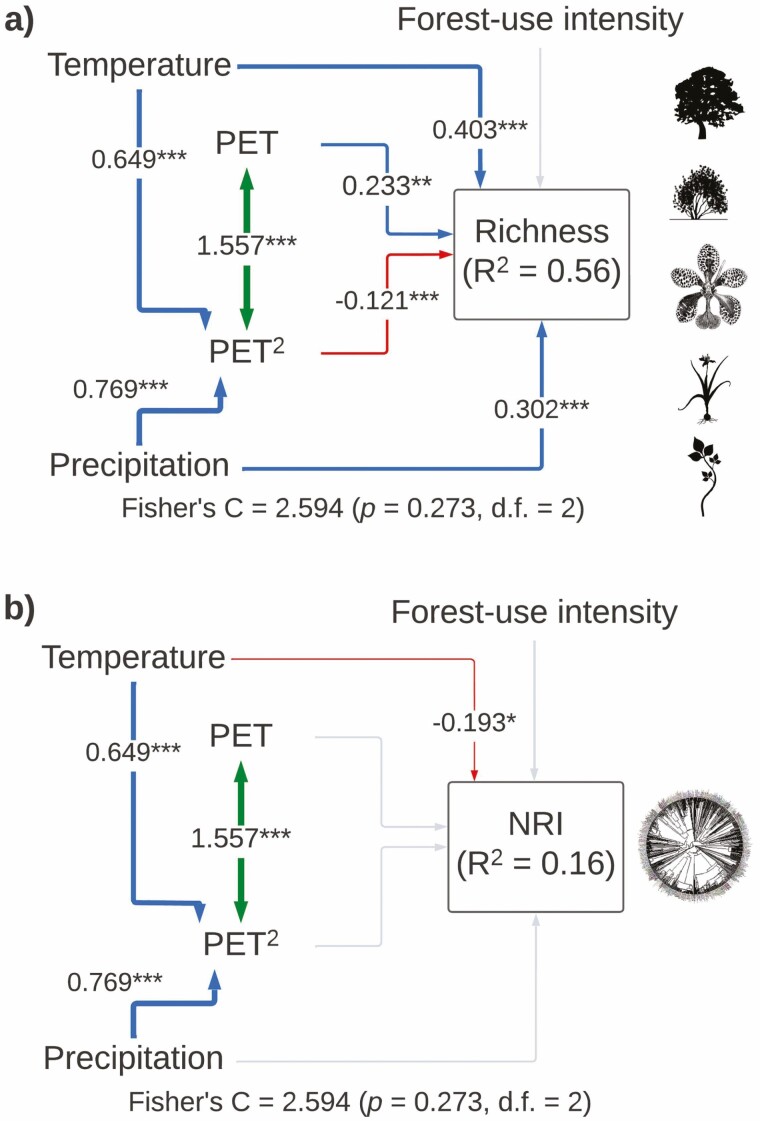
Structural equation models showing direct and indirect effects of predictor variables on (A) total species richness and (B) total net relatedness index (NRI). For all paths of the SEM, arrow width is proportional to the relative strength of standardized path coefficients. The coefficients are shown on the arrows, if they are not there it means that they were not significant. Significances are *P < 0.05, **P < 0.01 and ***P < 0.001. Silhouettes represent plant life forms from PhyloPic (phylopic.org).

Our SEM for total species richness explained 56 % of the variance and included all important paths (Fisher’s *C* = 2.594, *P*-value = 0.273, df = 2). The temperature was the most important variable influencing total species richness (total standardized effect = 0.403), followed by precipitation and a unimodal effect of PET (Fig. 3A; Table 1). Our model for NRI explained a low proportion of its variance (*r*^2^ = 0.16) and included all important paths (Fisher’s *C* = 2.594, *P*-value = 0.273, df = 2). The temperature was the only variable that influenced total NRI (total standardized effect = −0.195; Fig. 3B; Table 1).

**Table 1.** Total direct standardized effects of temperature, precipitation, potential evapotranspiration (PET), a quadratic term of potential evapotranspiration (PET^2^), and disturbance on species richness and near relatedness index (NRI) in our final path models. Significances are *P < 0.05, **P < 0.01 and ***P < 0.001.

Groups	Temperature	Precipitation	PET	PET^2^	Forest-use intensity
Richness	0.403***	0.302***	0.233***	−0.121***	
Trees	1.024***	0.522***	0.173*	−0.128**	−0.121*
Shrubs	0.269***				
Epiphytes	1.217***	0.912***	0.688***	−0.295***	
Herbs	−0.135**	0.245***	0.181**	−0.092*	
Lianas	1.532***				
NRI	−0.193*				
NRI shrubs	−0.268*				
NRI epiphytes	0.659**		0.432**	−0.204***	
NRI herbs	−0.308**	0.341**			

**Figure 4.**
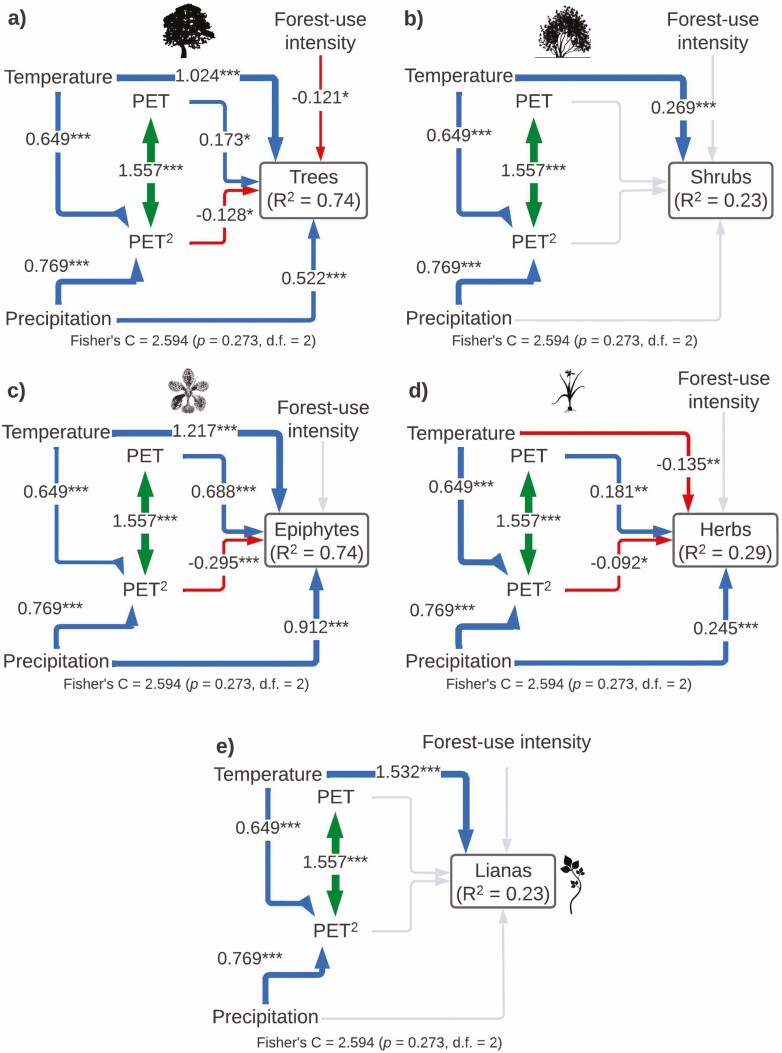
Structural equation models showing direct and indirect effects of predictor variables on species richness by plant life form (A: trees, B: shrubs, C: epiphytes, D: herbs and E: lianas). For all paths of the path model, arrow width is proportional to the relative strength of standardized path coefficients. The coefficients are shown on the arrows, if they are not there it means that they were not significant. Significances are *P < 0.05, **P < 0.01 and ***P < 0.001. Silhouettes represent plant life forms from PhyloPic (phylopic.org).

The SEM model for species richness of each plant life form included all the important paths according to the Global goodness-of-fit test (Fisher’s *C* = 2.594, *P*-value = 0.273, df = 2) and explained the variance of each group differently: trees (*r*^2^ = 0.74), shrubs (*r*^2^ = 0.23), epiphytes (*r*^2^ = 0. 74), herbs (*r*^2^ = 0.29) and lianas (*r*^2^ = 0.23; Fig. 4; Table 1). Tree richness was affected by a direct effect of temperature, precipitation and a unimodal influence of PET. Disturbance also had a negative direct effect on tree richness. Shrub richness was only influenced by a positive direct effect of temperature. Epiphyte richness was affected by a direct effect of temperature, precipitation and a unimodal influence of PET. Herb richness was affected negatively by temperature and positively by precipitation, whereas productivity had a non-linear (monotonic) direct effect on herb richness. Finally, liana richness showed a direct linear positive effect on temperature (Fig. 4; Table 1).

**Figure 5.**
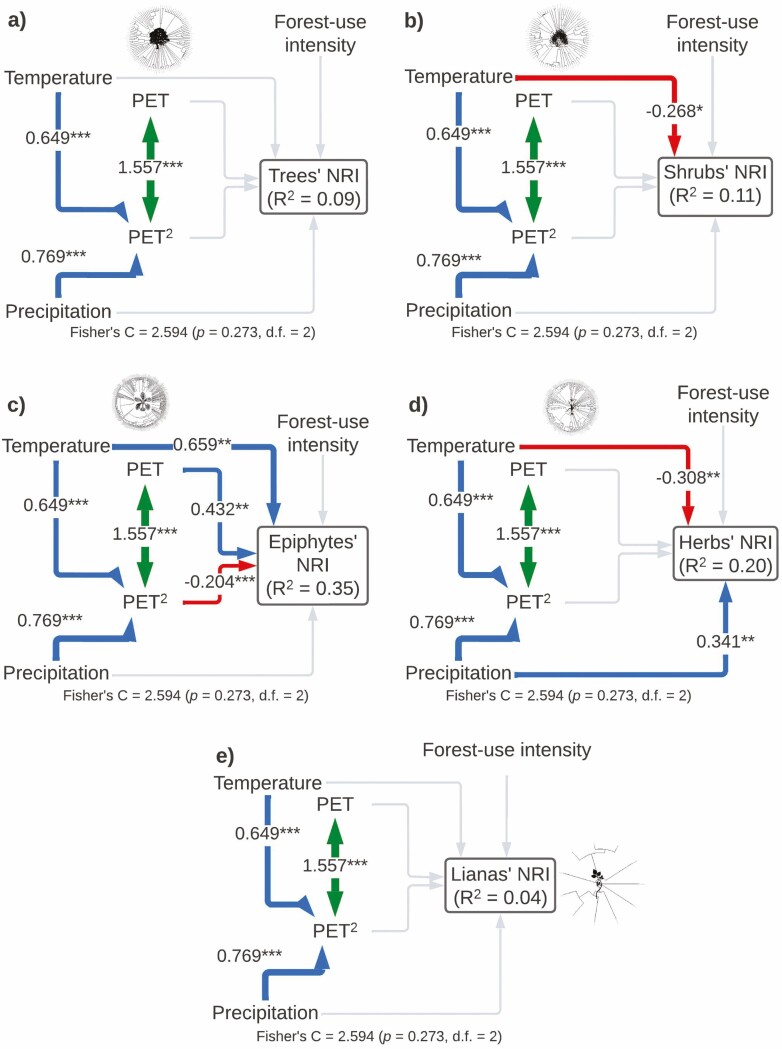
Structural equation models showing direct and indirect effects of predictor variables on near relatedness index by plant life form (A: trees, B: shrubs, C: epiphytes, D: herbs and E: lianas). For all paths of the path model, arrow width is proportional to the relative strength of standardized path coefficients. The coefficients are shown on the arrows, if they are not there it means that they were not significant. Significances are *P < 0.05, **P < 0.01 and ***P < 0.001. Silhouettes represent plant life forms from PhyloPic (phylopic.org).

The model for NRI of each plant life form included all the important paths according to the Global goodness-of-fit test (Fisher’s *C* = 2.594, *P*-value = 0.273, df = 2) and explained the variance of each group differently: trees (*r*^2^ = 0.09), shrubs (*r*^2^ = 0.11), epiphytes (*r*^2^ = 0.35), herbs (*r*^2^ = 0.20) and lianas (*r*^2^ = 0.04; Fig. 5; Table 1). NRI of shrubs was only affected by a negative linear direct effect of temperature. Epiphyte’s NRI was affected by a positive direct linear effect of temperature and a monotonic non-linear direct effect of PET. Herb’s NRI was affected negatively by temperature and positively by precipitation. Finally, the NRI of trees and lianas was not affected by any of the included variables (Fig. 5; Table 1).

## Discussion

As hypothesized by Humboldt more than 200 years ago, the climate is a strong determinant of plant species richness along elevational gradients. Humboldt proposed that climate influences the distribution of species along an elevational gradient and even proposed the mechanism of how this relation works (Rahbek *et al*. 2019). He stated that fluidity is essential for life (see Humboldt *et al*. 1850), noting that temperature and water have a decisive dynamical relationship to explain diversity along gradients. In general, biological and biochemical processes require water in a liquid state, which is controlled by temperature (Schrodt *et al*. 2019). From these assertions, the hypothesis of water–energy dynamics emerges, which explains the patterns of species richness spatially (O’Brien *et al*. 1998; O’Brien 2006).

Indeed, here we found that the species richness of all plant life forms was influenced directly by temperature. Precipitation and PET were also important for most plant life forms (except for shrubs and lianas), whereas anthropogenic disturbance only influenced trees. Moreover, we found that temperature also influenced the phylogenetic structure of all plants and most of the life forms (except for trees and lianas) along elevation, precipitation influenced the NRI of only herbs, and only in the case of the phylogenetic structure of epiphytes, there was also an influence of PET.

### Relationship between elevation and environmental variables

Elevational gradients are like natural laboratories that have been used to capture environmental and climatic variation and evaluate their effect on diversity patterns (Graham *et al*. 2014). Elevational gradients have a methodological advantage by presenting a wide climatic variation in a short geographical distance. In summary, an elevational gradient makes it possible to evaluate an environmental gradient of great magnitude, in a smaller and more controlled area, something that Humboldt himself elucidated in his works (Schrodt *et al*. 2019).

However, according to Rahbek *et al*. (2019), elevation is a variable that *per se* does not have any effect on biodiversity, but it is the climatic variations associated with elevational changes in a gradient that have a direct effect on diversity patterns. In this sense, one of the environmental variables associated with elevation is temperature. The temperature has an inverse relationship with elevation this is because solar radiation heats the ground and this in turn the atmosphere. Since there is less land surface at high elevations, less heat is transferred to the atmosphere during the day (Whiteman 2000). Another environmental variable related to elevation is precipitation which usually increases with elevation. Though, this pattern depends on other climatological variables (wind direction, continentality, latitude, etc.; Whiteman 2000). In some tropical mountains (as in our study area), the highest precipitation has been observed at intermediate elevations with a marked decrease at higher elevations (Leuschner 2000).

Potential evapotranspiration as a surrogate of energy (O’Brien 1998) decreases with elevation (Bhattarai and Vetaas 2003). In our study area, we found that this variable followed a U-shaped pattern with elevation. This may be due to the influence of other climatological variables as in the case of precipitation (Whiteman 2000). Finally, forest-use intensity is an anthropogenic variable which depends on multiple factors not necessarily related to elevation *per se*. Nevertheless, there is evidence that areas with more complex topography (ruggedness) have lower forest-use intensity than low elevations and mountain bases (Elsen *et al*. 2020).

### Temperature

We found support for our first hypothesis: (a) plant species richness shows an increase with temperature. Temperature is one of the major predictors of species richness according to the species-energy hypothesis (Allen *et al*. 2007), influencing plant physiology (Pausas and Austin 2001). We found a similar pattern of species richness showing a positive linear relationship with temperature for all plant life forms (except for herbs). In the case of trees, previous studies along elevational gradients showed that temperature is the main predictor of species richness (Toledo-Garibaldi and Williams-Linera 2014; Sharma *et al*. 2019; Monge-González *et al.* 2020). In the case of shrubs, they are adapted to warm climates and thus sensitive to temperature changes (Vázquez and Givnish 1998; Bautista-Bello *et al.* 2019). In the case of epiphytes, there is a positive effect between high environmental humidity and optimal temperature (Krömer *et al*. 2005, 2013). In the case of lianas, they are of tropical origin and thus adapted to warm climates, with the highest richness found in humid and seasonal tropical lowland forests (Parthasarathy 2015).

In contrast to the other plant life forms, we found that herb richness follows a negative linear relationship with temperature. This can result from the competitive advantage of herbs in low-temperature sites over other plant groups (Vázquez and Givnish 1998), given their ecological characteristics such as higher morphological flexibility in their adaptations to low temperatures and having shorter generation periods than trees, which enables them to diversify more rapidly at higher elevations (Gomez-Diaz 2017).

The temperature was the only variable shaping the NRI of all plant life forms, showing phylogenetic overdispersion with an increase in temperature. This reinforces our hypothesis (d) of higher clustering at high elevations and hypothesis (e) with more distantly related species being more common at elevated temperatures, due to high interspecific competition (Coyle *et al*. 2014).

For shrubs and herbs, temperature negatively influenced their phylogenetic structure, with a trend towards phylogenetic clustering at colder sites. This may be related to the presence of some shrubs (e.g. *Acaena elongata*, *Baccharis conferta*, *Barkleyanthus salicifolius*, *Fuchsia microphylla* and *Pernettya prostrata*) and herb species (e.g. *Alchemilla vulcanica*, *Muhlenbergia macroura*, *Penstemon gentianoides*, *Senecio callosus* and *Arenaria reptans*) of Nearctic origin that are adapted to the cold climates of high elevations. The opposite pattern was found for epiphytes, with a strong positive effect of temperature on their phylogenetic structure, with warmer sites associated with showing higher phylogenetic clustering. This pattern can be related to strong selection pressures due to extreme conditions of temperature and drought over these plants (Shapcott *et al*. 2015).

### Precipitation

We did find the influence of precipitation on total species richness, providing evidence to support hypothesis (b) high species richness under higher precipitation. Also, we found that precipitation influenced the species richness of trees, epiphytes and herbs because the lack of this resource can function as a strong limiting factor for those life forms (Gentry 1982; Kreft *et al*. 2004). The herb’s phylogenetic structure was positively affected by precipitation, with closely related species being present under higher precipitation conditions. This may be related to the adaptation of some groups or genera within the three main families of angiosperm herbs to humid conditions such as Asteraceae (specifically hygrophilous), Cyperaceae (*Carex*) and Poaceae (Gomez-Diaz 2017).

### Potential evapotranspiration

It is well known that PET influences species richness (Currie 1991). Accordingly, we found that energy translated into primary productivity influenced plant species richness along our elevational gradient. These findings agree with previous studies on an extensive tropical elevational gradient (Kilimanjaro; Peters *et al*. 2016) and the Himalayas (Vetaas *et al*. 2019). For the tree, epiphyte and herb richness, we found a hump-shaped relationship with PET. In the case of trees, our results corroborate those of Bhattarai and Vetaas (2003) showing a significant unimodal response to PET, which is consistent with the water–energy dynamics model (O’Brien *et al*. 1998; 2000).

In the case of epiphytes, our results corroborate those of Vetaas *et al*. (2019) showing a richness peak at the subtropical/warm temperate zones transition. Most epiphytes will have a maximum richness in the tropical–subtropical biome at mid-elevations (Gentry and Dodson 1987; Krömer *et al*. 2005), which is also corroborated by the tropical niche conservatism hypothesis (Vetaas *et al*. 2019). Finally, PET had a unimodal effect on the epiphyte’s phylogenetic structure with clustering at mid-values of PET and overdispersion at the extremes of the gradient of PET.

### Forest-use intensity

Humboldt’s historical descriptions of elevational gradients have been used to model and predict the effect of modern environmental change on species migration (Morueta-Holme *et al*. 2015). Humboldt even mentioned the effect of human disturbance on the current distribution of plants and how these changes can be as strong as those related to environmental variables. Unfortunately, very few elevational studies incorporate the anthropic effect as an environmental factor that can influence plant diversity, so it is essential in current biogeography to relate this effect to climate (see Peters *et al*. 2016; Schrodt *et al*. 2019).

We found that species richness was not related to forest-use intensity, providing insufficient evidence for hypothesis (c). This is due to the response of only trees to this anthropogenic disturbance. Therefore, there is no general pattern for total species richness. Our analyses revealed that forest-use intensity had a direct negative effect on tree richness, suggesting that high forest-use intensity may reduce the individual and special richness of these plant life forms. Indeed, the effects of forest-use intensity on tree species richness are widely documented (Newbold *et al*. 2015).

Moreover, we did not find any support for our last hypothesis on forest-use intensity negatively affecting the phylogenetic structure of plants. According to Brunbjerg *et al*. (2012), the linkage between anthropogenic disturbance and phylogenetic clustering of species may be a general pattern. However, this hypothesis is based on the prediction that disturbance inflicts an environmental filter on species based on their functional traits, thereby filtering lineages (Ding *et al*. 2012; Zhang *et al*. 2014). Yet, the phylogenetic structure among species of angiosperm plants along our elevational gradient was unrelated to forest-use intensity (Fig. 3). This finding indicates that the response to such a disturbance among plants may be randomly distributed across the plants’ phylogenetic tree (Zhang *et al*. 2014). Overall, these findings refute the universality of reduced phylogenetic clustering following disturbance (Zhang *et al*. 2014).

## Conclusion

Given the current global environmental crisis, an integrative biogeographically oriented vision is necessary, which is based on Humboldt’s method of collecting copious amounts of data and inferring and explaining causality instead of simply describing patterns (SEM approach vs. traditional model selection approach). Overall, we found that species richness and phylogenetic structure were mostly influenced by temperature, with precipitation and PET also being important for species richness and the phylogenetic structure of some groups.

Our findings revealed that species richness was: (i) positively affected by temperature (support for hypothesis a); (ii) related to precipitation (evidence for hypothesis b) and (iii) not related to forest-use intensity (not enough evidence for hypothesis c). In the case of plant phylogenetic structure, we found that the phylogenetic structure had: (iv) an overdispersed structure at higher temperatures (support for hypothesis d); (v) a clustered structure at lower temperatures (support for hypothesis e) and (vi) any effect due to forest-use intensity (no support for our hypothesis f).

Alexander von Humboldt noted that the types of habitats and the number of species varied predictably with changes in latitude and elevation. In his explorations of tropical mountains, Humboldt suggested that the climate is a crucial factor in determining plant species richness along elevational gradients. Honouring the work of Humboldt and continuing his legacy demands more research to understand the causes behind elevational diversity gradients. Finally, it is necessary to integrate species richness as well as the phylogenetic structure and ‘deconstruct’ the pattern by looking at life forms to have a better insight into the processes shaping biodiversity along elevational gradients.

## Supporting Information

The following additional information is available in the online version of this article—

Figure S1. Patterns of the studied climatic variables (MAT = mean annual temperature, MAP = mean annual precipitation, and PET = potential evapotranspiration) along the elevational gradient of the Cofre de Perote mountain, Veracruz, Mexico.

Figure S2. Patterns of species richness of the studied life forms (all, trees, shrubs, epiphytes, herbs, lianas) along the elevational gradient of the Cofre de Perote mountain, Veracruz, Mexico.

Figure S3. Patterns of near relatedness index (NRI) of the studied life forms (all, trees, shrubs, epiphytes, herbs, lianas) along the elevational gradient of the Cofre de Perote mountain, Veracruz, Mexico.

plac056_suppl_Supplementary_Figures

plac056_suppl_Supplementary_Data_S1

plac056_suppl_Supplementary_Data_S2

plac056_suppl_Supplementary_Data_S3

plac056_suppl_Supplementary_Data_S4

## Data Availability

All data and the R code to reproduce the results are provided as **Supporting Information**.

## Notes

**Populations & Communities. Chief Editor:** Jean Burns
