# Supplementary material for: Humboldt’s legacy: explaining the influence of environmental factors on the taxonomic and phylogenetic diversity of angiosperms along a Neotropical elevational gradient
Source: AoB Plants. 2022 Nov 7;15(1):plac056. doi: 10.1093/aobpla/plac056 (PMC9840209; doi:10.1093/aobpla/plac056)
Supplement: plac056_suppl_Supplementary_Figures [file plac056_suppl_supplementary_figures.pdf]

Supplementary information

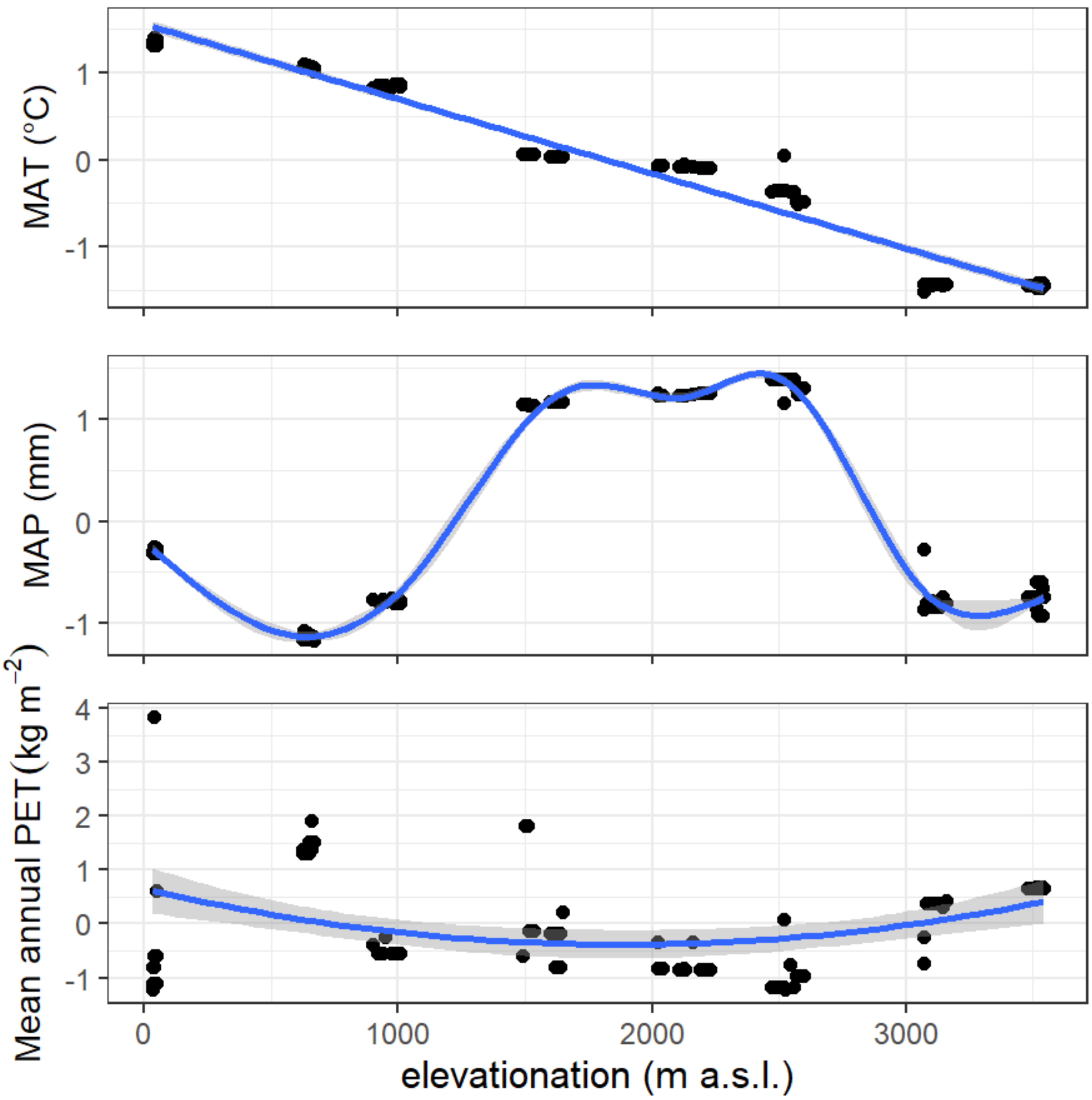

Figure S1. Patterns of the studied climatic variables (MAT = mean annual temperature, MAP = mean annual precipitation, and PET = potential evapotranspiration) along the elevational gradient of the Cofre de Perote mountain, Veracruz, Mexico.

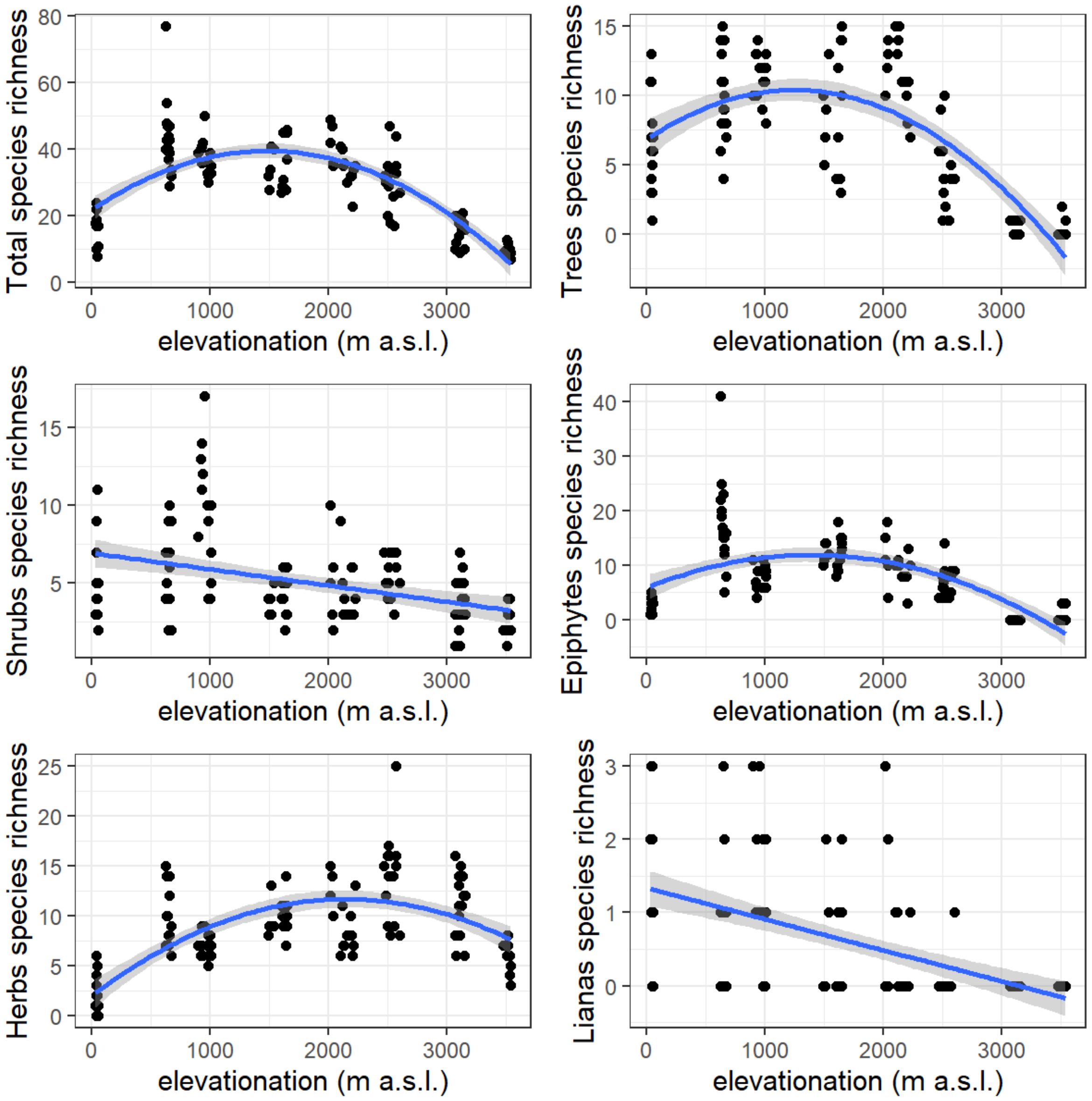

Figure S2. Patterns of species richness of the studied life forms (all, trees, shrubs, epiphytes, herbs, lianas) along the elevational gradient of the Cofre de Perote mountain, Veracruz, Mexico.

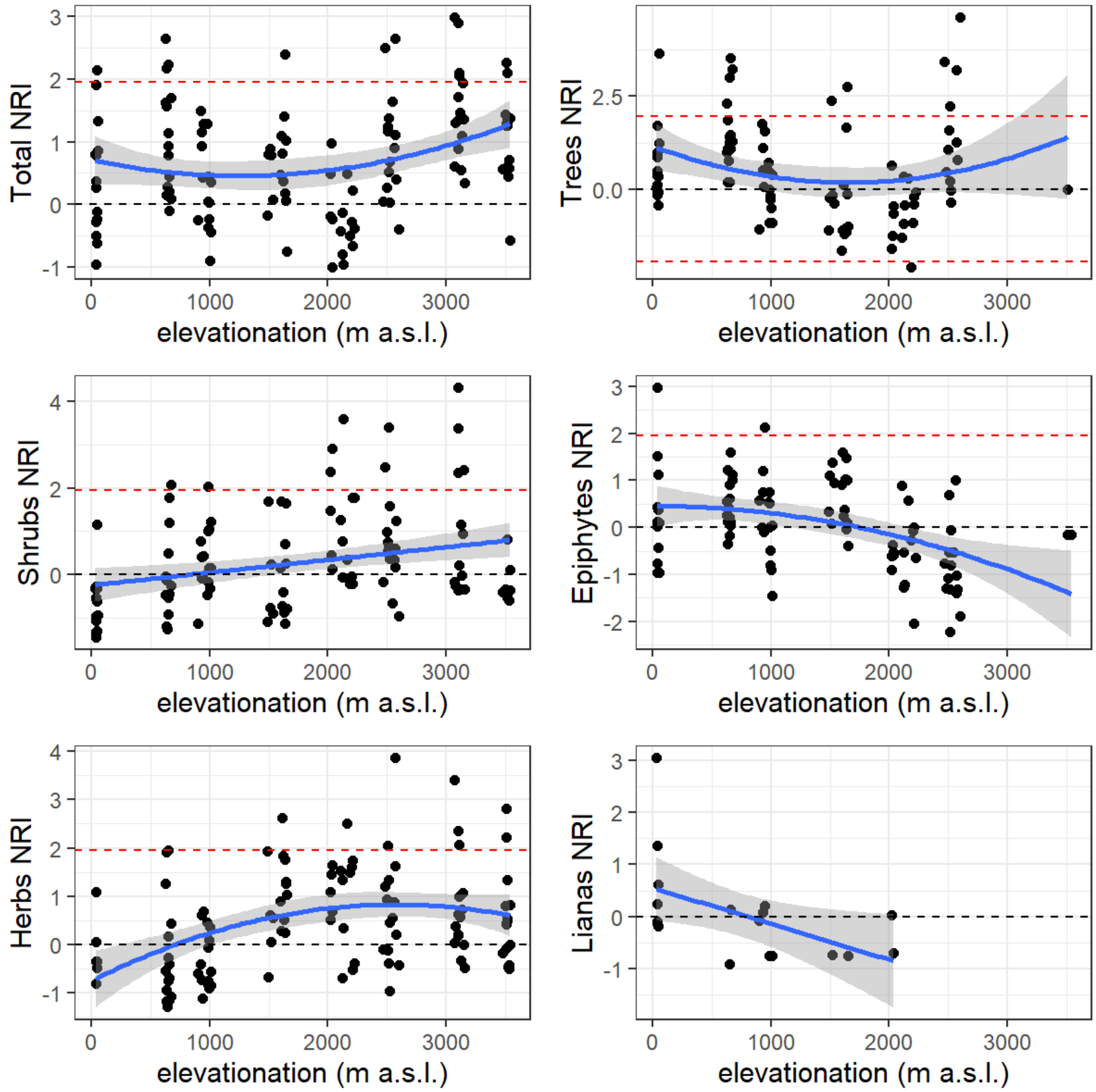

Figure S3. Patterns of near relatedness index (NRI) of the studied life forms (all, trees, shrubs, epiphytes, herbs, lianas) along the elevational gradient of the Cofre de Perote mountain, Veracruz, Mexico.
